# Editorial

**Published:** 1847-04

**Authors:** 


### THE MEDICAL EXAMINER.

PHILADELPHIA, APRIL, 1847.

### APPLICATION OF REMEDIES TO THE INFLAMED SURFACE IN CHRONIC LARYNGITIS.

We observe in certain of the Medical journals somewhat intemperate discussions in regard to the first employment of topical remedies to the inflamed surface in chronic laryngitis; and great credit is given to Dr. Green of New York for having been an originator of the practice. Dr. Green's work has not been sent to us, and therefore we know nothing of the facts and arguments which he employs, but no reader of the productions of the day ought to be ignorant, that the works on the Practice of Medicine published in this country in 1842 contained details of the method advised by MM. Trousseau and Belloq, to whom the main credit is due for the introduction of topical agents into the larynx. The first edition of Dr. Dunglison's Practice of Medicine, published in 1842, and therefore written some time previously, refers to it, and so does Dr. Bell's edition of Stokes's Lectures, published in the same year. An injudicious exercise of friendship is often most detrimental to the cause which it is intended to support, and we think that Dr. Green has had ample cause to exclaim—"Save me from my friends!"

### INSENSIBILITY DURING SURGICAL OPERATIONS.

In a former number, we mentioned that a patent had been taken out by two gentlemen in Boston, for the discovery of a mode of rendering patients insensible to the pain caused by severe surgical operations. It was then spoken of as a "*compound gas*," and since called "*letheon.*" Misled at the time by the name given to the substance employed, we conjectured that it was an ethereal solution of some narcotic. It is now ascertained by numerous experiments, that sulphuric ether alone posseses the virtues claimed for the "letheon" or "compound gas," and hence much of the mystery with which the subject was sought to be en-enveloped is dispelled, and as we believe it is generally conceded, both in this country and Europe, that the patent is invalid, the repugnance we felt towards any extended notice of the matter is removed, and we shall from time to time notice the experiments and observations made in various parts of the world, confirmatory or otherwise of the good effects of this extraordinary agent. In our "Record" will be found an account of numerous experiments made with the ether in France, by some of the most distinguished surgeons, which may be regarded as an epitome of our present knowledge on the subject, so nearly does it accord with the recorded observations made in this country and in Great Britain. Notwithstanding the many favourable reports, we cannot divest ourselves of the belief that the employment of an agent which is capable of rendering a person unconscious of pain during the performance of a severe surgical operation, must, when carried to that extent, be fraught with danger—danger the more to be dreaded because it cannot be estimated, owing to variations in the dose, and the different susceptibilities of those to whom it is administered. Bad effects have indeed been repeatedly witnessed, and even death, in a few instances. The sagacious Velpeau, we perceive, looks upon it with great distrust.

---

### JEFFERSON MEDICAL COLLEGE.

An account of the Commencement of this College, held on the 25th inst., will be found on another page, from which it will be seen that the Degree of Doctor of Medicine was conferred on one hundred and eighty-one gentlemen—the largest number ever graduated at any institution in the United States.

---

### PENNSYLVANIA COLLEGE.

The class of this Institution during the last Session we understand numbered ninety-five ; and the Graduates thirty-four, including two honorary Degrees.

---

### FRANKLIN MEDICAL COLLEGE.

Five gentlemen were graduated at this new College at its recent Commencement. Of the number of the class we are not informed.

---

### CITY OF NEW YORK MEDICAL SCHOOLS.

By the papers we are informed that the class at the University School during the last Session numbered four hundred and ten, and the number graduated was one hundred and twenty-three ; and that at the College of Physicians and Surgeons fifty-one graduated.

ALBANY MEDICAL COLLEGE.

From a copy of the "Catalogue and Circular" of this Institution which we have received, we learn that the class of the last Session, (1846—7) numbered 100; and that of that number, 30 graduated at the conclusion of the term. The class of the previous Session numbered 115, and the Graduates 42.

TRANSYLVANIA UNIVERSITY.

We derive from the printed Catalogue of the Transylvania University, that the class in attendance on the lectures during the past winter numbered 182; and the graduating class 68, including 4 honorary degrees. The class of the preceding Session (1845—6) numbered 171, and the graduates 64; showing a small increase for the past Session.

